# Diacridinium tetra­kis(thio­cyanato-κ*S*)platinate(II)

**DOI:** 10.1107/S1600536810002485

**Published:** 2010-01-23

**Authors:** Kwang Ha

**Affiliations:** a School of Applied Chemical Engineering, The Research Institute of Catalysis, Chonnam National University, Gwangju 500-757, Republic of Korea

## Abstract

The asymmetric unit of the title compound, (C_13_H_10_N)_2_[Pt(NCS)_4_], contains a protonated acridine molecule and one half of a [Pt(NCS)_4_]^2−^ anion. In the complex anion, the Pt^II^ ion is located on an inversion centre and is four-coordinated in a slightly distorted square-planar environment by four S atoms from four thio­cyanate ligands. The compound displays numerous inter­molecular π–π inter­actions between six-membered rings, with a shortest centroid–centroid distance of 3.682 (3) Å. The component ions inter­act by means of inter­molecular N—H⋯N hydrogen bonds.

## Related literature

For related acridinium compounds, see: Hafiz (2006 ▶); Veldhuizen *et al.* (1997 ▶). For the crystal structures of [*M*(NCS)_4_]^2−^ [*M* = Pt(II), Pd(II)] complexes, see: Aoki *et al.* (1999 ▶); Deplano *et al.* (2004 ▶); Rohde *et al.* (2000 ▶).
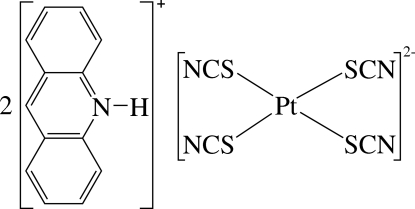


## Experimental

### 

#### Crystal data

(C_13_H_10_N)_2_[Pt(NCS)_4_]
*M* *_r_* = 787.85
Monoclinic, 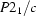

*a* = 6.8358 (8) Å
*b* = 11.9833 (15) Å
*c* = 17.737 (2) Å
β = 93.618 (2)°
*V* = 1450.0 (3) Å^3^
*Z* = 2
Mo *K*α radiation
μ = 5.16 mm^−1^
*T* = 293 K
0.11 × 0.10 × 0.10 mm

#### Data collection

Bruker SMART 1000 CCD diffractometer
Absorption correction: multi-scan (*SADABS*; Bruker, 2001 ▶) *T*_min_ = 0.463, *T*_max_ = 1.000
8278 measured reflections
2968 independent reflections
2003 reflections with *I* > 2σ(*I*)
*R*_int_ = 0.039

#### Refinement

*R*[*F* ^2^ > 2σ(*F* ^2^)] = 0.040
*wR*(*F* ^2^) = 0.068
*S* = 1.03
2968 reflections
187 parameters
H-atom parameters constrained
Δρ_max_ = 0.89 e Å^−3^
Δρ_min_ = −0.50 e Å^−3^

### 

Data collection: *SMART* (Bruker, 2007 ▶); cell refinement: *SAINT* (Bruker, 2007 ▶); data reduction: *SAINT*; program(s) used to solve structure: *SHELXS97* (Sheldrick, 2008 ▶); program(s) used to refine structure: *SHELXL97* (Sheldrick, 2008 ▶); molecular graphics: *ORTEP-3* (Farrugia, 1997 ▶) and *PLATON* (Spek, 2009 ▶); software used to prepare material for publication: *SHELXL97*.

## Supplementary Material

Crystal structure: contains datablocks global, I. DOI: 10.1107/S1600536810002485/hy2274sup1.cif

Structure factors: contains datablocks I. DOI: 10.1107/S1600536810002485/hy2274Isup2.hkl

Additional supplementary materials:  crystallographic information; 3D view; checkCIF report

## Figures and Tables

**Table 1:** Selected bond lengths (Å)

Pt1—S1	2.3236 (17)
Pt1—S2	2.3254 (17)

**Table 2:** Hydrogen-bond geometry (Å, °)

*D*—H⋯*A*	*D*—H	H⋯*A*	*D*⋯*A*	*D*—H⋯*A*
N3—H3⋯N1^i^	0.86	1.97	2.829 (6)	177

Symmetry code: (i) 
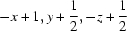
.

**Fig. 1.**
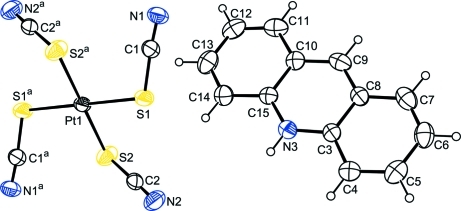
The structure of the title compound, with displacement ellipsoids drawn at the 30% probability level. [Symmetry code: (a) 2-x, 1-y, 1-z.]

**Fig. 2.**
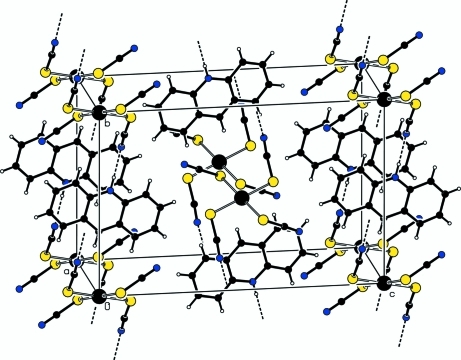
View of the unit-cell contents of the title compound. Hydrogen-bond interactions are drawn with dashed lines.

## supplementary crystallographic information

### Comment

The asymmetric unit of the title compound contains a protonated acridine cation and one half of a [Pt(NCS)_4_]^2-^ anionic complex (Fig. 1). In the complex, the Pt^II^ ion is located on an inversion centre at the special position (1, 1/2, 1/2) and is four-coordinated in a slightly distorted square-planar environment by four S atoms from four NCS^-^ ligands. The Pt—S bond lengths are nearly equivalent [2.3236 (17) and 2.3254 (17) Å] (Table 1). The *cis* S—Pt—S bond angles are 88.82 (6) and 91.18 (6)°. The thiocyanate anions are almost linear displaying S—C—N bond angles of 175.7 (6) and 176.9 (6)°. The S atoms coordinate to the Pt atom with nearly tetrahedral Pt—S—C bond angles of 105.9 (2) and 104.4 (2)°. The compound displays numerous intermolecular π–π interactions between six-membered rings, with a shortest centroid–centroid distance of 3.682 (3) Å. The component ions interact by means of intermolecular N—H···N hydrogen bonds (Fig. 2 and Table 2).

### Experimental

To a solution of K_2_PtCl_6_ (0.2002 g, 0.412 mmol) in H_2_O (20 ml) was added KNCS (0.3998 g, 4.114 mmol) and refluxed for 1 h. After cooling of the reaction mixture to room temperature, acridine (0.1479 g, 0.825 mmol) was added and refluxed for 3 h. The precipitate obtained was separated by filtration, washed with H_2_O and dried at 50 °C, to give an orange powder (0.1894 g). Crystals suitable for X-ray analysis were obtained by slow evaporation from a MeOH solution.

### Refinement

H atoms were positioned geometrically and allowed to ride on their respective parent atoms [C—H = 0.93, N—H = 0.86 Å and *U*_iso_(H) = 1.2*U*_eq_(C, N)].

### Crystal data

**Table:**

(C_13_H_10_N)_2_[Pt(NCS)_4_]	*F*(000) = 768
*M**_r_* = 787.85	*D*_x_ = 1.804 Mg m^−^^3^
Monoclinic, *P*2_1_/*c*	Mo *K*α radiation, λ = 0.71073 Å
Hall symbol: -P 2ybc	Cell parameters from 720 reflections
*a* = 6.8358 (8) Å	θ = 2.3–20.2°
*b* = 11.9833 (15) Å	µ = 5.16 mm^−^^1^
*c* = 17.737 (2) Å	*T* = 293 K
β = 93.618 (2)°	Block, orange
*V* = 1450.0 (3) Å^3^	0.11 × 0.10 × 0.10 mm
*Z* = 2	

### Data collection

**Table:**

Bruker SMART 1000 CCD diffractometer	2968 independent reflections
Radiation source: fine-focus sealed tube	2003 reflections with *I* > 2σ(*I*)
graphite	*R*_int_ = 0.039
φ and ω scans	θ_max_ = 26.4°, θ_min_ = 2.3°
Absorption correction: multi-scan (*SADABS*; Bruker, 2001)	*h* = −8→8
*T*_min_ = 0.463, *T*_max_ = 1.000	*k* = −14→14
8278 measured reflections	*l* = −11→22

### Refinement

**Table:**

Refinement on *F*^2^	Primary atom site location: structure-invariant direct methods
Least-squares matrix: full	Secondary atom site location: difference Fourier map
*R*[*F*^2^ > 2σ(*F*^2^)] = 0.040	Hydrogen site location: inferred from neighbouring sites
*wR*(*F*^2^) = 0.068	H-atom parameters constrained
*S* = 1.02	*w* = 1/[σ^2^(*F*_o_^2^) + (0.0175*P*)^2^] where *P* = (*F*_o_^2^ + 2*F*_c_^2^)/3
2968 reflections	(Δ/σ)_max_ < 0.001
187 parameters	Δρ_max_ = 0.89 e Å^−^^3^
0 restraints	Δρ_min_ = −0.50 e Å^−^^3^

### Fractional atomic coordinates and isotropic or equivalent isotropic displacement parameters (Å^2^)

**Table:**

	*x*	*y*	*z*	*U*_iso_*/*U*_eq_	
Pt1	1.0000	0.5000	0.5000	0.05422 (12)	
S1	0.8814 (3)	0.44333 (14)	0.38044 (9)	0.0807 (5)	
S2	1.2773 (2)	0.56944 (17)	0.44661 (10)	0.0862 (6)	
N1	0.6764 (7)	0.2432 (4)	0.3972 (3)	0.0747 (16)	
N2	1.1409 (9)	0.6690 (5)	0.3098 (3)	0.095 (2)	
C1	0.7597 (8)	0.3255 (5)	0.3932 (3)	0.0609 (16)	
C2	1.1910 (9)	0.6278 (5)	0.3653 (4)	0.0686 (19)	
N3	0.2844 (5)	0.5161 (3)	0.0610 (3)	0.0497 (11)	
H3	0.2917	0.5852	0.0740	0.060*	
C3	0.2417 (6)	0.4939 (5)	−0.0132 (3)	0.0454 (12)	
C4	0.2091 (7)	0.5803 (5)	−0.0660 (4)	0.0569 (15)	
H4	0.2199	0.6546	−0.0513	0.068*	
C5	0.1610 (8)	0.5522 (6)	−0.1395 (4)	0.0666 (18)	
H5	0.1365	0.6083	−0.1751	0.080*	
C6	0.1481 (8)	0.4401 (7)	−0.1621 (4)	0.0730 (18)	
H6	0.1161	0.4233	−0.2126	0.088*	
C7	0.1808 (8)	0.3563 (6)	−0.1124 (4)	0.0708 (19)	
H7	0.1722	0.2826	−0.1288	0.085*	
C8	0.2283 (7)	0.3802 (5)	−0.0354 (3)	0.0515 (14)	
C9	0.2682 (7)	0.2991 (5)	0.0195 (4)	0.0611 (17)	
H9	0.2664	0.2245	0.0051	0.073*	
C10	0.3109 (7)	0.3256 (5)	0.0957 (3)	0.0521 (14)	
C11	0.3508 (8)	0.2451 (5)	0.1531 (4)	0.0675 (18)	
H11	0.3496	0.1696	0.1411	0.081*	
C12	0.3904 (9)	0.2780 (6)	0.2253 (4)	0.077 (2)	
H12	0.4196	0.2249	0.2626	0.092*	
C13	0.3877 (8)	0.3919 (6)	0.2445 (4)	0.0752 (19)	
H13	0.4116	0.4126	0.2948	0.090*	
C14	0.3509 (8)	0.4722 (5)	0.1915 (3)	0.0614 (17)	
H14	0.3488	0.5471	0.2052	0.074*	
C15	0.3163 (7)	0.4401 (5)	0.1159 (3)	0.0473 (13)	

### Atomic displacement parameters (Å^2^)

**Table:**

	*U*^11^	*U*^22^	*U*^33^	*U*^12^	*U*^13^	*U*^23^
Pt1	0.0612 (2)	0.04675 (18)	0.0540 (2)	−0.00893 (17)	−0.00227 (16)	0.00275 (19)
S1	0.1100 (14)	0.0712 (11)	0.0591 (11)	−0.0381 (11)	−0.0097 (10)	0.0064 (10)
S2	0.0712 (12)	0.1146 (15)	0.0721 (12)	−0.0280 (11)	−0.0007 (10)	0.0130 (12)
N1	0.090 (4)	0.052 (3)	0.081 (4)	−0.013 (3)	−0.005 (3)	0.006 (3)
N2	0.105 (5)	0.113 (5)	0.069 (4)	−0.034 (4)	0.008 (4)	0.014 (4)
C1	0.071 (4)	0.054 (4)	0.056 (4)	−0.001 (3)	−0.011 (3)	0.003 (3)
C2	0.072 (5)	0.068 (5)	0.067 (5)	−0.029 (4)	0.020 (4)	−0.017 (4)
N3	0.043 (2)	0.048 (3)	0.058 (3)	0.004 (2)	0.007 (2)	−0.004 (3)
C3	0.032 (3)	0.053 (3)	0.053 (3)	0.000 (3)	0.011 (2)	−0.006 (3)
C4	0.042 (3)	0.060 (4)	0.069 (4)	0.001 (3)	0.005 (3)	0.003 (4)
C5	0.043 (3)	0.092 (5)	0.066 (5)	0.006 (3)	0.010 (3)	0.011 (4)
C6	0.062 (4)	0.097 (5)	0.060 (4)	0.006 (4)	0.009 (4)	−0.015 (5)
C7	0.054 (4)	0.073 (5)	0.086 (5)	−0.005 (3)	0.015 (4)	−0.027 (4)
C8	0.039 (3)	0.058 (4)	0.060 (4)	−0.005 (3)	0.015 (3)	−0.013 (3)
C9	0.044 (3)	0.048 (4)	0.093 (5)	−0.003 (3)	0.021 (4)	−0.011 (4)
C10	0.042 (3)	0.056 (4)	0.060 (4)	0.006 (3)	0.009 (3)	−0.001 (3)
C11	0.053 (4)	0.051 (4)	0.099 (6)	0.010 (3)	0.012 (4)	0.011 (4)
C12	0.073 (5)	0.081 (5)	0.078 (5)	0.010 (4)	0.014 (4)	0.027 (5)
C13	0.062 (4)	0.102 (6)	0.062 (4)	0.009 (4)	0.006 (4)	0.008 (5)
C14	0.060 (4)	0.063 (4)	0.061 (4)	0.005 (3)	0.003 (3)	−0.003 (3)
C15	0.038 (3)	0.048 (3)	0.057 (4)	0.003 (3)	0.009 (3)	0.001 (3)

### Geometric parameters (Å, °)

**Table:**

Pt1—S1	2.3236 (17)	C6—H6	0.9300
Pt1—S2	2.3254 (17)	C7—C8	1.414 (7)
S1—C1	1.661 (6)	C7—H7	0.9300
S2—C2	1.676 (7)	C8—C9	1.390 (7)
N1—C1	1.143 (6)	C9—C10	1.400 (7)
N2—C2	1.135 (7)	C9—H9	0.9300
N3—C15	1.341 (6)	C10—C11	1.417 (7)
N3—C3	1.357 (6)	C10—C15	1.417 (7)
N3—H3	0.8600	C11—C12	1.350 (8)
C3—C4	1.403 (7)	C11—H11	0.9300
C3—C8	1.419 (7)	C12—C13	1.408 (8)
C4—C5	1.367 (7)	C12—H12	0.9300
C4—H4	0.9300	C13—C14	1.358 (7)
C5—C6	1.403 (9)	C13—H13	0.9300
C5—H5	0.9300	C14—C15	1.402 (7)
C6—C7	1.345 (8)	C14—H14	0.9300
			
S1^i^—Pt1—S1	180.0	C6—C7—H7	120.0
S1^i^—Pt1—S2	91.18 (6)	C8—C7—H7	120.0
S1—Pt1—S2	88.82 (6)	C9—C8—C7	123.9 (6)
S1^i^—Pt1—S2^i^	88.82 (6)	C9—C8—C3	118.1 (5)
S1—Pt1—S2^i^	91.18 (6)	C7—C8—C3	118.0 (6)
S2—Pt1—S2^i^	180.0	C8—C9—C10	122.5 (5)
C1—S1—Pt1	105.9 (2)	C8—C9—H9	118.8
C2—S2—Pt1	104.4 (2)	C10—C9—H9	118.8
N1—C1—S1	175.7 (6)	C9—C10—C11	123.9 (6)
N2—C2—S2	176.9 (7)	C9—C10—C15	117.6 (6)
C15—N3—C3	125.9 (5)	C11—C10—C15	118.4 (6)
C15—N3—H3	117.1	C12—C11—C10	120.1 (6)
C3—N3—H3	117.1	C12—C11—H11	120.0
N3—C3—C4	121.2 (5)	C10—C11—H11	120.0
N3—C3—C8	117.6 (5)	C11—C12—C13	120.5 (6)
C4—C3—C8	121.2 (5)	C11—C12—H12	119.8
C5—C4—C3	118.3 (6)	C13—C12—H12	119.8
C5—C4—H4	120.9	C14—C13—C12	121.7 (6)
C3—C4—H4	120.9	C14—C13—H13	119.2
C4—C5—C6	121.0 (6)	C12—C13—H13	119.2
C4—C5—H5	119.5	C13—C14—C15	118.7 (6)
C6—C5—H5	119.5	C13—C14—H14	120.6
C7—C6—C5	121.5 (6)	C15—C14—H14	120.6
C7—C6—H6	119.2	N3—C15—C14	121.2 (5)
C5—C6—H6	119.2	N3—C15—C10	118.3 (5)
C6—C7—C8	120.0 (6)	C14—C15—C10	120.6 (6)
			
S2—Pt1—S1—C1	145.5 (2)	C7—C8—C9—C10	−178.7 (5)
S2^i^—Pt1—S1—C1	−34.5 (2)	C3—C8—C9—C10	3.3 (8)
S1^i^—Pt1—S2—C2	−140.0 (2)	C8—C9—C10—C11	179.5 (5)
S1—Pt1—S2—C2	40.0 (2)	C8—C9—C10—C15	−1.2 (8)
C15—N3—C3—C4	179.4 (4)	C9—C10—C11—C12	−179.9 (5)
C15—N3—C3—C8	0.3 (7)	C15—C10—C11—C12	0.8 (8)
N3—C3—C4—C5	−178.1 (5)	C10—C11—C12—C13	1.6 (9)
C8—C3—C4—C5	0.9 (7)	C11—C12—C13—C14	−1.8 (10)
C3—C4—C5—C6	−1.2 (8)	C12—C13—C14—C15	−0.4 (9)
C4—C5—C6—C7	0.5 (9)	C3—N3—C15—C14	−177.1 (5)
C5—C6—C7—C8	0.4 (9)	C3—N3—C15—C10	1.8 (7)
C6—C7—C8—C9	−178.7 (5)	C13—C14—C15—N3	−178.2 (5)
C6—C7—C8—C3	−0.7 (8)	C13—C14—C15—C10	2.9 (8)
N3—C3—C8—C9	−2.8 (7)	C9—C10—C15—N3	−1.3 (7)
C4—C3—C8—C9	178.1 (5)	C11—C10—C15—N3	178.0 (5)
N3—C3—C8—C7	179.0 (4)	C9—C10—C15—C14	177.6 (5)
C4—C3—C8—C7	0.0 (7)	C11—C10—C15—C14	−3.1 (7)

Symmetry codes: (i) −*x*+2, −*y*+1, −*z*+1.

### Hydrogen-bond geometry (Å, °)

**Table:**

*D*—H···*A*	*D*—H	H···*A*	*D*···*A*	*D*—H···*A*
N3—H3···N1^ii^	0.86	1.97	2.829 (6)	177

Symmetry codes: (ii) −*x*+1, *y*+1/2, −*z*+1/2.
